# Development of a Novel, Fully-Automated Genotyping System: Principle and Applications

**DOI:** 10.3390/s121216614

**Published:** 2012-12-03

**Authors:** Shun-ichi Suzuki, Mariko Komori, Mitsuharu Hirai, Norio Ureshino, Shinya Kimura

**Affiliations:** 1 ARKRAY Inc., Kyoto 602-0008, Japan; E-Mails: suzukishu@arkray.co.jp (S.S.); komorim@arkray.co.jp (M.K.); hiraim@arkray.co.jp (M.H.); 2 Division of Haematology, Respiratory Medicine, and Oncology, Faculty of Medicine, Saga University, Saga 849-8501, Japan; E-Mail: ureshino-n@koseikan.jp; 3 Saga Prefectural Hospital Koseikan, Saga 840-8571, Japan

**Keywords:** automated genotyping system, pharmacogenetic analysis, single nucleotide polymorphism, i-densy^™^, Qprobe, mutation biased PCR, personalized medicine

## Abstract

Genetic testing prior to treatment, pharmacogenetic analysis, is key to realizing personalized medicine which is a topic that has attracted much attention recently. Through the optimization of therapy selection and dosage, a reduction in side effects is expected. Genetic testing has been conducted as a type of pharmacogenetic analysis in recent years, but it faces challenges in terms of cost effectiveness and its complicated procedures. Here we report on the development of a novel platform for genetic testing, the i-densy^™^, with the use of quenching probe system (QP-system) as principle of mutant detection. The i-densy^™^ automatically performs pre-treatment, PCR and detection to provide the test result from whole blood and extracted DNA within approximately 90 and 60 min, respectively. Integration of all steps into a single platform greatly reduces test time and complicated procedures. An even higher-precision genetic analysis has been achieved through the development of novel and highly-specific detection methods. The applications of items measured using the i-densy^™^ are diverse, from single nucleotide polymorphism (SNP), such as *CYP2C19* and *UGT1A1*, to somatic mutations associated with cancer, such as *EGFR*, *KRAS* and *JAK2*. The i-densy^™^ is a useful tool for optimization of anticancer drug therapy and can contribute to personalized medicine.

## 1. Introduction

Recently, great advances in genomic research into drug sensitivity have broadened the use of genetic information in clinical practice. Moreover, the information and tools necessary to identify important genetic associations are widely available. The increasing availability of genetic tests in clinical laboratories is also facilitating the application of pharmacogenomic testing in patient care. In particular, a great deal of development into individual gene typing of single nucleotide polymorphisms (SNPs) and somatic mutations of various cancers has been conducted [1–4]. The introduction of pharmacogenomics, allows for medications which are based on genome information resulting in improved safety and efficacy. The promise of “personalized medicine” is therefore steadily progressing toward becoming a reality [5,6].

These advances in genomic research reveal that gene polymorphism and genetic mutation are related to the therapeutic effect of many drugs, requiring a simple genetic test method. To meet this demand, we have developed a platform for genetic testing called the “i-densy^™^”. The i-densy^™^ can complete fully automated gene polymorphism analysis from whole blood and extracted DNA within about 90 and 60 min, respectively. Novel and highly-specific detection systems enable the highly-accurate detection of minor mutations derived from cancer cells. These techniques could contribute to in-house analysis of gene polymorphisms and genetic mutations. Herein we introduce the principle, features, and applications of the i-densy^™^.

## 2. Measurement Principle of a Guanine Quenching Probe (QProbe)

### 2.1. QProbe

**Figure 1.**
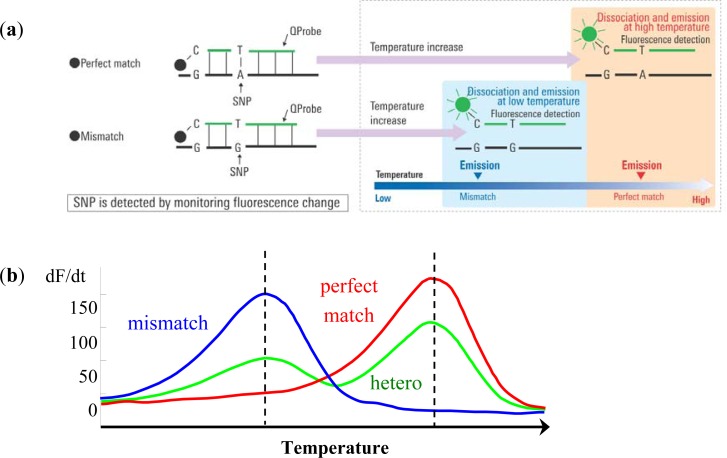
Measurement principle—Quenching Probe (QProbe). (**a**) QProbe quenches when hybridizing to the target nucleic acid. By detecting the change of fluorescence, it is possible to detect the existence of the target sequence. (**b**) A typical detection result using QProbe.

The Quenching probe (QProbe, Nippon Steel Kankyo Engineering Co., Ltd., Tokyo, Japan) is a fluorescent probe in which a fluorescent substance is bound to cytosine on the terminal region of the probe, which becomes quenched upon hybridization with a complementary strand. With increasing temperature, the duplex unravels at a temperature (Tm) related to the strength of the bond between the QProbe and the complementary chain, at which point the fluorescence intensity recovers (Figure 1(a)). Tm analysis is a technique to determine the degree of complementation between the QProbe and target nucleic acid by measuring the change in fluorescence intensity with increasing temperature [7].

QProbes are designed to be short in order to widen differences in Tm values between mismatch and perfect match, which leads to increased detection sensitivity. However, if the length of QProbe is too short, mismatch peaks may become very small. Moreover, primers are designed so that the length of PCR products at the target region will be around 100 bp, and thereby enabling brief and effective PCR.

Briefly put, PCR is performed at the target region. The reagent contains QProbes with complementary sequences of the target region including either wild type or mutant type sequences. As the temperature is decreased following PCR, the probe and amplified product are hybridized regardless of whether a mismatch is present or not. Then, using a gradual temperature increase, the loosely bound mismatch sequences and probes detach and fluorescence is emitted. When the temperature is increased further, perfect match sequences and probes will detach and fluorescence will increase. This method using QProbe is based on analysis of the probe DNA melting curve (QP-system). The detected fluorescence is differentiated and converted into peaks. In case mismatch sequences are included, peaks are detected at a lower temperature than that of perfect match sequences. Therefore, SNPs or somatic mutations can be detected by the difference in temperature and fluorescence through the use of QProbe (Figure 1(b)) [8,9].

### 2.2. Mutation-Biased PCR (MBP) and the QP-System (MBP-QP System)

**Figure 2.**
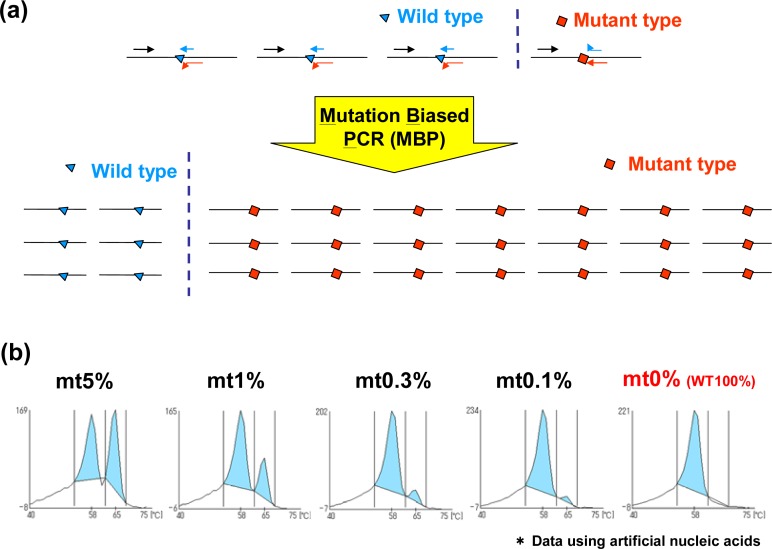
Measurement principle—Mutation biased PCR (MBP). (**a**) The primers for wild and mutant types are mixed with plasmid DNA, which leads to high specificity because each primer can be competitively hybridized to wild type and mutant type sequences. In addition, the length of primer for mutant type is longer than that for wild type, and annealing temperature was optimized to the mutant primer, resulting in higher amplification efficiency for the mutant type compared to the wild type. (**b**) Measurement results of the detection of T790M mutation in *EGFR* exon 20 using MBP-QP system.

We have developed MBP-QP system which is about 10 times more sensitive than a traditional QP-system [10]. For MBP, the 3′ terminal region of primer is established at the mutation site, and the primers for wild type and mutant type are used for amplification. This method offers high specificity because each primer can be competitively hybridized to wild type and mutant type sequences. In addition, the length of the primer for the mutant is longer than that for wild type, and annealing temperature is designed to be optimum to mutant primer, resulting in higher efficiency of amplification with the mutant compared to that of the wild type sequence (Figure 2(a)). Furthermore, the correlation between the proportion of the mutant form and the value of the fluorescence intensity is detected using the QP-system. The sensitivity of mutation detection using this method was tested using plasmids carrying *EGFR* exon 20 with or without T790M mutation, and the detection of T790M mutation can be around 0.3% mutant plasmids (Figure 2(b)). Peaks are detected at higher temperature in the presence of T790M mutation because QProbes are designed to perfectly match T790M mutation. It has been reported that *c-kit* mutation, a novel adverse prognostic factor of acute myeloid leukemia (AML) with t(8;21)(q22;q22) translocation, or the minor population of *EGFR* T790M in plasma DNA can be detected with high sensitivity by the use of MBP-QP system [11]. MBP-QP system is suitable for sensitive detection of point mutations.

### 2.3. Wild Inhibition PCR (WIP) and QP-System (WIP-QP System)

**Figure 3.**
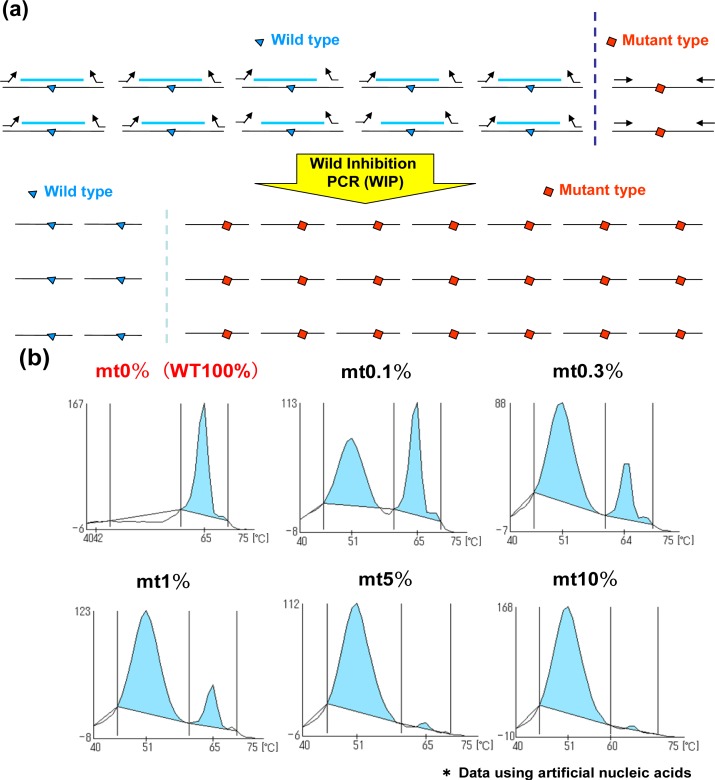
Measurement principle—Wild Inhibition PCR (WIP). (**a**) DNA fragments from the wild type as well as primers for the wild and mutant types are mixed with plasmid DNA. Amplification of wild type sequence is inhibited by this DNA fragment. (**b**) Measurement results of the detection of in-frame deletion in *EGFR* exon 19 using WIP-QP system.

In addition to the MBP-QP system, the WIP-QP system is also sensitive and can be applied to the detection of in-frame deletion in *EGFR* exon 19 [12]. For WIP, wild type DNA fragments are established at the in-frame deletion site, and this DNA fragment, forward primer and reverse primer are used for amplification. The DNA fragment is intensely bound to wild-type sequence in the presence of wild type sequence, while the amplification of the wild type sequence is inhibited. Meanwhile, the DNA fragment is not tightly bound to the mutant sequence, in-frame deletion site, resulting in higher efficiency of amplification of mutant type compared to the wild type (Figure 3(a)). When two plasmids carrying *EGFR* exon19 with or without the in-frame deletion are mixed in different ratios, the detection of in-frame deletion in exon 19 can be around 0.1% mutant plasmids (Figure 3(b)). Peaks are detected at lower temperature in the presence of in-frame deletion in exon 19 because QProbes are designed to perfectly match wild type exon 19 at the in-frame deletion site. WIP-QP system is suitable for highly sensitive detection of deletion mutations.

## 3. Features of the i-densy^™^

**Figure 4.**
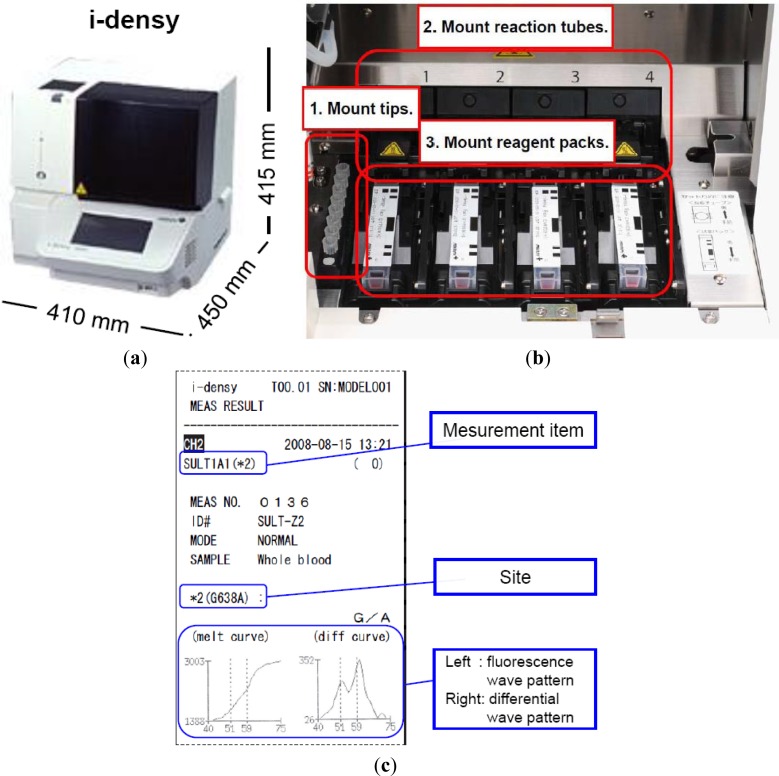
i-densy^™^—Fully integrated and automatic gene typing system. (**a**) i-densy^™^ has a compact design: 410 mm (width) by 450 mm (depth) by 415 mm (height). (**b**) Before measurement, the necessary number of tips, reaction tubes, and reagent packs are put in place. The instrument contains 4 independently programmable reaction sites. (**c**) When measurement is complete, measurement results are automatically printed out.

As indicated above, we have developed a platform for genetic testing called the i-densy^™^, which can complete fully automated gene polymorphism analysis from whole blood in approximately 90 minutes, and compared it with an existing test method. The i-densy^™^ has a more compact design: 410 mm (width) × 450 mm (depth) × 415 mm (height) (Figure 4(a)). The system allows fully integrated automatic gene-typing from sample pretreatment to gene amplification and signal detection. The equipment incorporates computer-free analysis so that measurement results can also be analyzed within a single system. The working time has been reduced by using a newly developed technique for sample pretreatment that does not require DNA extraction. Following placement of the reagent pack and the sample, gene-typing results are available in about 90 min.

The features and specifications of the i-densy are as follows:

**Key features:**
- Pretreatment, amplification, and detection are all automated.
- Automated testing procedures setup using barcodes on the disposable reagent.
- Closed system for reagents.
- Rapid processing from preparation to detection.
  <Within 90 min>
- Four independently programmable reaction sites.
- 3-color optical detection for each site.

**Specifications**
- Color LCD with touch screen
- Data storage: up to 25 users, and 500 measurements per user
- EMC compliant
- Computer linkage via USB/Ethernet

Prior to measurement, the necessary number of tips, reaction tubes and reagent packs are put in place. The equipment contains four independently programmable reaction sites (Figure 4(b)). Reagent packs, reaction tubes, and tips are all included in one package. The reagent pack contains a reagent for pretreatment, amplification and detection. Sample is applied into the sample well. In this system, whole blood, oral swabs or purified DNA can be used as SNP-typing materials. When a blood sample is used, the sample well is filled with not less than 70 μL of whole blood. In addition, when a purified DNA sample is used, just 4 μL of purified DNA sample is required, and added directly to the reaction tube. Measurement is begun by simply pressing the start key, the i-densy^™^ then automatically performs pre-treatment, PCR and detection to provide the test result. After measurement is complete, results can be printed out. Previous results can also be printed (Figure 4(c)). Moreover, the use of i-densy Pack Universal, a reagent pack filled with amplification reagents and pre-treatment reagents except primers and probes, enables measurement of any item using the i-densy^™^. Thus, the i-densy^™^ has broad utility.

## 4. Applications of the i-densy^™^

In collaboration with a number of medical experts, a number of applications have been developed (Table 1).

### 4.1. SNP Detection

#### 4.1.1. CYP2C19

Cytochrome P450 is a family of the body’s more powerful detoxification enzymes. It is known that *CYP2C19* is one of the cytochrome P450 families and its genotype is associated with changes in drug effects [13]. Moreover, it has been reported that CYP2C19*2 and CYP2C19*3 influences the blood kinetics of the proton pump inhibitors (PPIs), which are closely related to their efficacy [8,14]. The fast metabolic type has been reported to reduce the healing rapidity of peptic ulcers, the eradication rate of *Helicobacter pylori* and the curative effect of gastroesophageal reflux disease. Therefore, the dose setting and choice of PPIs should be made according to the *CYP2C19* polymorphism [15].

A recent study suggested that the results obtained by the QP-system were completely identical to those examined by direct sequencing (DS), indicating the accuracy of the QP-system [8]. In addition, QP-system using the i-densy^™^ enables a one-step process and provides results within about 90 min. The DS method usually provides the results in about a week as it requires several steps, including the extraction of DNA from a sample, the amplification of the target region on the extracted DNA using PCR, and the sequencing of the target SNP in the amplified product. Thus, it is clear that a QP-system using the i-densy^™^ is accurate, as with DS method, and is superior to the DS method in terms of convenience.

#### 4.1.2. UGT1A1

Irinotecan is a camptotencin-derived anticancer drug. At present, irinotecan is highly regarded worldwide and essential for therapy of colon cancer epecially in the West. Uridine diphosphate-glucuronosyl transferase 1A1 (*UGT1A1*) is of particularly note as to its relationship with toxicity. UGT1A1*6 and UGT1A1*28, the genetic polymorphisms of *UGT1A1*, are associated with adverse effects to irinotecan. Patients who are homozygous for UGT1A1*28 had a nine times higher risk of developing grade 4 neutropenia. For Caucasians, the detection of UGT1A1*28 is more important than UGT1A1*6 because UGT1A1*6 is rarely observed in them. Meanwhile, the allele frequency of UGT1A1*28 is lower in Asians than in Caucasians, and grade 3–4 hematologic toxicity associated with UGT1A1*6 polymorphisms in Asians [16,17]. The UGT1A1*6 allele is found in 20–30% of Asians, including Japanese [8,18]. Thus, there is a need to diagnose the presence not only UGT1A1*28 but also UGT1A1*6 in Asian populations [19]. It has been reported that results obtained by QP-system matched with the DS method. Moreover, both UGT1A1*6 and UGT1A1*28 are concomitantly measured using one reagent pack [8].

### 4.2. Somatic Mutation Associated with Cancer

#### 4.2.1. EGFR

Epidermal growth factor receptor (*EGFR*) tyrosine kinase inhibitors are widely used to treat lung adenocarcinomas, especially in non-small cell lung cancer (NSCLC), with *EGFR*-activating mutations [20,21]. The susceptibility to *EGFR* tyrosine kinase inhibitors (TKIs) validates the fundamental dependence of NSCLC tumors on *EGFR* mutations for maintaining the malignant phenotype [22]. All of the somatic activating *EGFR* mutations involve the adenosine triphosphate (ATP)-binding pocket in the receptor TK domain, which is the binding site for the TKIs, erlotinib and gefitinib. These mutations lead to a ligand-independent activation of TK.

Somatic mutations of *EGFR* gene are found in exon 18 through 21 of the TK domain. The most common mutations are the in-frame deletions in exon 19 and a substitution of lysine for arginine mutation at amino acid position 858 (L858R) in exon 21 [23]. These deletions in exon 19 constantly include amino acid residues 747 to 749, and account for about 44% of all *EGFR* activating mutations. Meanwhile, L858R accounts for about 41% of all *EGFR* activating mutations [22]. It is well known that the efficacy of targeted therapies such as gefitinib or erlotinib with NSCLC patients depends on the presence of *EGFR* activating mutations including in-frame deletion in exon 19 or L858R in exon 21 [22–25].

However, many patients acquire resistance to EGFR-TKI, which occurs within 9.5 to 14 months [26–28]. A second *EGFR* mutation, substitution of threonine 790 with methionine (T790M), was detected in approximately 50% of the patients who had acquired resistance to EGFR-TKI [29]. T790M activates the wild type EGFR and increases the affinity for ATP on *EGFR* activating mutations resulting in resistance to EGFR-TKI [30]. Because the T790M mutation is not the only cause of acquired resistance to EGFR-TKI, it is also important to determine the type of resistance in each patient to decide on appropriate treatment. A monitoring system is critical to achieving such a specific level of treatment, and a noninvasive mutation detection system is desirable given the difficulty in obtaining tissue specimens during disease progression.

It has been reported that the MBP-QP system is simple, sensitive, and can accurately monitor the clinical course by measuring T790M mutation [31]. In this study, DNA was extracted from 200 uL of patient’s or healthy volunteer’s plasma in order to investigate the detection of T790M mutation in peripheral blood. T790M mutation of *EGFR* was then measured using MBP-QP system. T790M mutation was also measured using PNA-LNA PCR clamp method, Cycleave method and allele-specific oligonucleotide PCR (AS-PCR) method. Among 19 patients with acquired resistance to EGFR-TKI, the number of patients tested positive for T790M mutation were 10 (53%) using the Mutation Biased PCR (MBP) method, three (16%) using the PNA-LNA PCR method and five (26%) using the Cycleave method. As a result, the MBP-QP system, when compared with the other mutation detection systems, can be seen as an ideal noninvasive monitoring system for detecting T790M mutation in plasma samples.

#### 4.2.2. KRAS/BRAF

The *KRAS* gene encodes a GTP-binding protein that contributes to cell proliferation and tumor progression. Mutations of *KRAS* are active, and GTP-bound forms of RAS and hot spots are present in codon 12 and 13 [32]. *KRAS* mutations are observed in tumors of the pancreas, colon, and lung [33]. *KRAS* mutations have recently been reported to be markers of resistance for EGFR-TKI and monoclonal antibodies against EGFR, cetuximab and panitumumab [32–36].

BRAF is one of the kinase types that most frequently mutate in human tumors. In melanoma, colon and thyroid cancers, the tumor types with highest frequency of *BRAF* mutation, a single nucleotide substitution resulting in a gulutamic acid for valine substitution within the kinase domain at codon 600 (V600E), accounts for the majority of cases [37]. This mutation results in elevated basal kinase activity, the activation of the ERK pathway and cellular transformation [38]. Moreover, it is reported that patients who carried *BRAFV600E* mutations did not respond to EGFR inhibition using cetuximab or panitumumab [35,38]. Therefore, *KRAS* and *BRAF* mutations are promising targets for cancer detection and screening.

Akagi *et al.* reported that *KRAS* mutation was measured concomitantly with *BRAF* mutation using the QP system, and that *KRAS* and *BRAF* mutations can be measured by use of depleted DNA extracted from paraffin-embedded tissue [39].

#### 4.2.3. JAK2

A somatic point mutation at nucleotide 1849 in exon 14 of the *jak2* gene results in the substitution of valine to phenylalanine at codon 617 (*JAKV617F*). This mutation has been identified in most polycythemia vera (PV) patients, approximately half of essential thrombocythemia (ET) and chronic idiopathic myelofibrosis with extramedullary hematopoiesis (CIMF) patients, and in a substantial population of other chronic myeloproliferative disease (CMPD) patients [40].

*JAK2V617F* is a gain-of-function mutation that stimulates JAK2 tyrosine kinase activity, leading to transformation of myeloid cells [41]. The early detection of *JAK2V617F* is important because patients carrying this mutation have a higher incidence of leukemic transformation compared with CMPD patients who do not have this mutation. *JAK2V617F* might also be a therapeutic target. Therefore, it is crucial that this mutation is accurately and rapidly detected.

It has been reported that using the QP-system, the *JAK2V617F* mutation was detected in 25 of 42 CMPD patients’ samples, while DS method failed to detect *JAK2V617F* in seven of those 25. The presence of *JAK2V617F* mutation in these seven samples was confirmed by allele-specific PCR. These results show that QP-system is more sensitive than DS method for diagnoses of CMPDs [9].

## 5. Possibility of the i-densy^™^

Growing evidence has recently demonstrated the profound impact of particular genetic mutations on the pathogenesis as well as diagnosis and treatment in various cancers. For example, mutations in the ABL kinase domain greatly impair the effect of the drug imatinib mesylate, an inhibitor of ABL tyrosine kinase, against chronic myeloid leukemia [42]. Thus, rapid and convenient detection of genetic mutations in individual patients will become increasingly important. The i-densy^™^ can theoretically be adapted to all mutations simply by exchanging reagent cartridges which contain specific PCR primers and QProbes designed for each mutation. Thus, the i-densy^™^ may become a useful tool in the field of molecular targeting therapy.

In conclusion, with its high sensitivity, convenience and speed, the i-densy^™^ will enable point-of-care testing in clinical laboratories and for patient-customized therapy for various cancers in the clinical field, such as decision of therapeutic strategy and selection of remedies, and could contribute to personalized medicine in the future.

## Figures and Tables

**Table 1.** Gene items measured with the i-densy^™^.

**Field**	**Gene**	**SNP/Mutation**	**Related Matters**
Cancer	EGFR	exon18 G719X	Drug efficacy prediction of gefitinib and erlotinib
		exon19 deletion	
		exon20 T790M	
		exon21 L858R	
	KRAS	codon12, 13	Drug efficacy prediction of cetuximab
	BRAF	V600E	
	SULT1A1	*2	Drug efficacy prediction of tamoxifen
	CYP2D6	*10	
	abl	T315I	Diagnosis of chronic myelogenous leukemia (CML), drug efficacy prediction of imatinib
		15 activate mutations	
	ABCG2	421C>A	
	ABCB1	1236C>T	Drug efficacy prediction of erlotinib
		2677G>T/A	
	EML4-ALK	Fusion gene	Drug efficacy prediction of crizotinib
	ALK	L1196M	
		C1156Y	
	JAK2	V617F	Diagnosis of myeloproliferative neoplasma
		Exon12 deletion	
	MPL	W515L/K	
Coagulation	PON1	Q192R	Drug efficacy prediction of clopidogrel
	CYP2C19	*2/*3	Drug efficacy prediction of many drugs such as clopidogrel
	CYP2C9	*3	Drug efficacy prediction of many drugs such as warfarin
	VKORC1	C1173T	
		−1639G>A	
HCV	IL28B	rs8099917(T/G)	Drug efficacy prediction of pegylated interferon
	ITPA	rs1127354	Prediction of adverse effect of ribavirin
Rheumatoid arthritis	IL-10	−819C>T	Related to antigen production
	MTHFR	C677T	Drug efficacy prediction of methotrexate
		A1298C	
	NAT2	*5/*6/*7	Drug efficacy prediction of antitubercular agent sulfasalazine and isoniazid
	TPMT	*3C	Drug efficacy prediction of mercaptopurine and azathioprine
Transplant	CYP3A4	*16	Drug efficacy prediction of many drugs such as tacrolimus
	CYP3A5	*3	
	ABCB1	3435C>T	Drug efficacy prediction of tacrolimus and cyclosporin
	ABCC2	−24C>T	
	ITPA	C94A	Drug efficacy prediction of azathioprine
	SLC28A1	G565A	Drug efficacy prediction of mizoribine
	SLC28A2	C65T	
	SLC28A3	A338G	
Resk prediction	ALDH2	*2	Prediction of alcohol sensitivity
	ADH2(ADH1B)	*2	
	β2AR	R16G	Prediction of basal metabolism
	β3AR	W64R	
	UCP1	A-3826G	
	FTO	rs9939609	
	ADRB3	rs4994	
	UCP1	rs1800592	
Prognostic factor	c-kit	D816-N822	Prognosis prediction of acute myelogenous leukemia (AML)
		D816H(CAC)	
		D816V(GTG)	
		N822K(AAG)	
